# Detecting Signatures of Criticality Using Divergence Rate

**DOI:** 10.3390/e27050487

**Published:** 2025-04-30

**Authors:** Tenzin Chan, De Wen Soh, Christopher Hillar

**Affiliations:** 1 Singapore University of Technology and Design, Singapore 487372, Singapore; tenzin_chan@mymail.sutd.edu.sg; 2 Information Systems Technology and Design, Singapore University of Technology and Design, Singapore 487372, Singapore; dewen_soh@sutd.edu.sg; 3 Algebraic, San Francisco, CA 94103, USA

**Keywords:** rate–distortion, optimal coding, criticality, phase transistion, Kullback–Leibler divergence, generalization

## Abstract

Oftentimes in a complex system it is observed that as a control parameter is varied, there are certain intervals during which the system undergoes dramatic change. In biology especially, these signatures of criticality are thought to be connected with efficient computation and information processing. Guided by the classical theory of rate–distortion (RD) from information theory, we propose a measure for detecting and characterizing such phenomena from data. When applied to RD problems, the measure correctly identifies exact critical trade-off parameters emerging from the theory and allows for the discovery of new conjectures in the field. Other application domains include efficient sensory coding, machine learning generalization, and natural language. Our findings give support to the hypothesis that critical behavior is a signature of optimal processing.

## 1. Introduction

Criticality has been a hallmark of many fundamental processes in nature. For instance, in classical work, Landau [1] and others [2] investigated critical phase transitions between discrete states of matter. More generally, this concept has been used to describe a complex system that is in sharp transition between two different regimes of behavior as one varies a control parameter. Often, a critical point borders ordered and disordered behavior, such as what occurs in the classical 2D Lenz–Ising spin model of statistical physics at special temperatures [3]. For instance, if a system is too hot, then, usually, disorder prevails, while, when it is too cold, it freezes into a low entropy configuration. The tipping points between such regimes have been the subject of much study in science.

In the recent literature, the notion of criticality [4,5,6] has grown to encompass a computational element [7,8], with an emphasis on how to apply it to understand information processing in natural dynamical systems such as organisms [9,10]. In these applications, it is frequently argued that optimal properties for computation emerge [11,12,13,14,15,16,17] when systems are critically poised at some special set of control parameters. Of particular excitement is how criticality can help neuroscientists understand the brain and where such signatures have been observed [18,19,20,21,22,23,24,25,26]. Another exciting area where criticality may be present is in large neural networks, which seem to generalize to certain problems at critical scales, training epochs, or dataset sizes [27,28].

Here, we leverage concepts from rate–distortion (RD) theory [29,30] to give insight into how normative principles governing a system [31], such as efficient coding [32,33], can lead to critical behavior. Consider a system *A* that evolves its internal steady-state dynamics through changes in a continuous control parameter *t*. The equilibrium behavior of *A* at *t* is modeled as a conditional distribution $p_{t}\left(y|x\right)$, which defines the probability that any given input *x* results in system state *y*. We say that *A* exhibits a critical phase transition about a special value $t^{*}$ of the control variable if $p_{t}\left(y|x\right)$ changes dramatically near this parameter setting.

**Figure 1:**
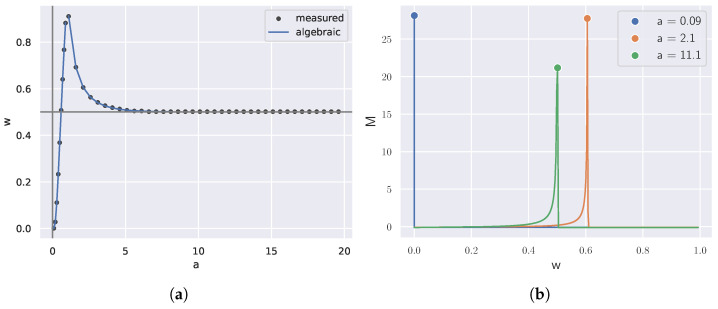
(**a**) Critical RD parameters $w^{*}=\exp\left(-\beta^{*}\right)$ determined mathematically from the theory (algebraic) match up exactly with significant peaks in the divergence rate (measured); see Section 3.1.1. (**b**) Plotting *M* vs. *w* for three different values of *a* shows the single peak in the divergence rate *M*.

**Figure 2:**
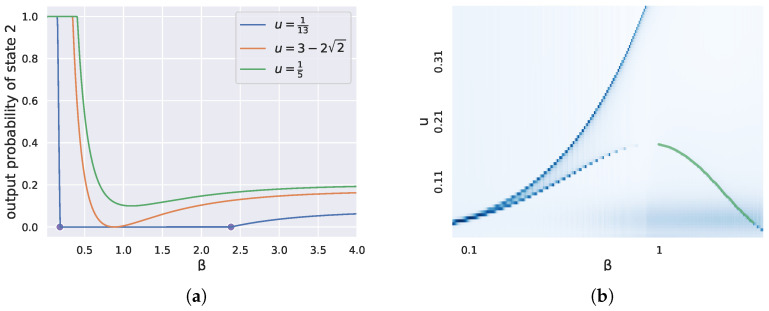
(**a**) Consider q=((1−u)/2,u,(1−u)/2) for a parameter *u* and a fixed distortion *d* given by (5). For each $u=1/13,3-2\sqrt{2},1/5$, we plot the output probabilities $p_{2}$ of the second state as a function of β, determined by the exact theory. The dots were determined from finding significant peaks in the divergence rate at u=1/13. Note that they coincided with the vanishing and re-emergence of state 2 ($p_{2}=0$). (**b**) We plotted a heatmap of the divergence rate as a function of *u* and β. We also plotted in green the theoretical calculation (7) that matched the lower-right part of the curve in the heatmap.

**Figure 3:**
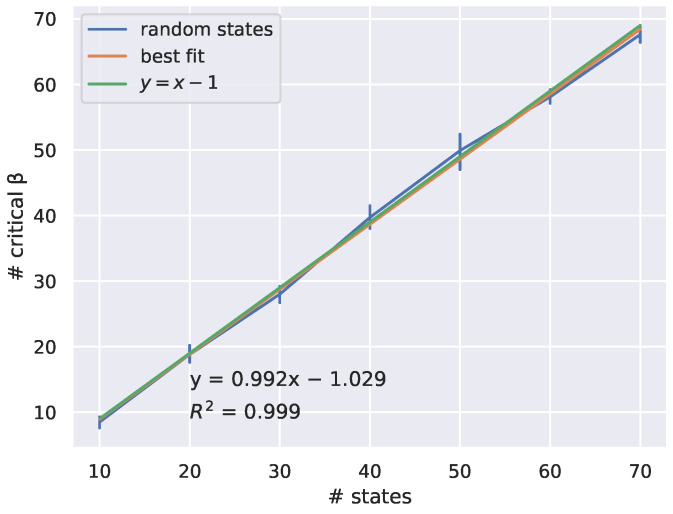
The number of peaks in divergence rate estimates the number # of critical β for a random RD problem with *n* states and is close to the line n−1 (coefficient of determination $R^{2}=0.999$).

To quantify the change at a given *t*, we average over inputs *x* the Kullback–Leibler divergence ($D_{KL}$) between distributions $p_{t}\left(y|x\right)$ and $p_{t+\Delta t}\left(y|x\right)$, with Δt small. We call this a divergence rate for the evolution of the system’s behavior along control parameter *t*. Given a sample of parameters *t*, we determine the significant peaks in the divergence rate, which we identify as critical control settings $t^{*}$ for the system. As we shall see in several examples, local maxima in the divergence rate seem to coincide with locations of phase transitions in a system (Figures 1–3). Intruigingly, this measure can uncover higher-dimensional manifolds of criticality (Figure 2b). This is the equivalent of finding the local maxima in the rate of change in evolving information-theoretic measures [34].

**Figure 4:**
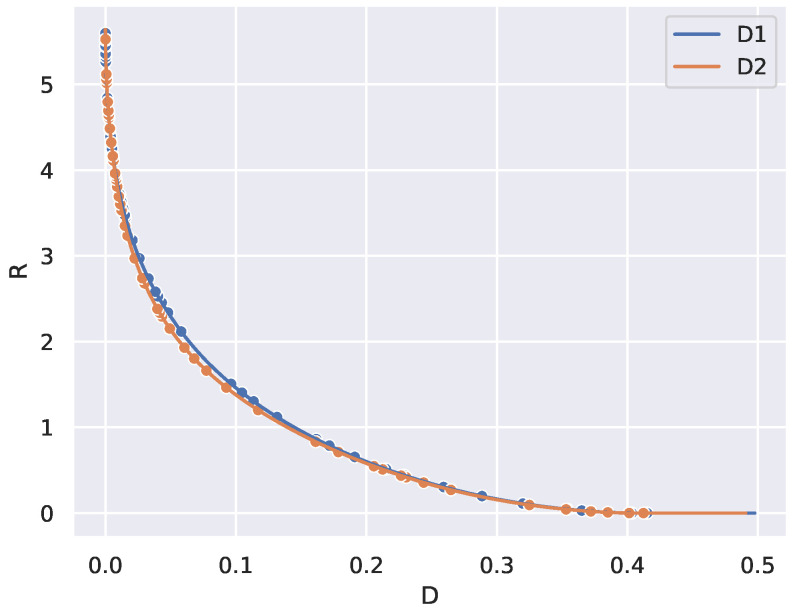
Taking two random distortion matrices D1 and D2 with entries drawn independently and uniformly in [0,1] gives two RD curves on n=50 states that look similar but whose critical points differ (points indicated along the curves), as estimated from divergence rate maxima.

**Figure 5:**
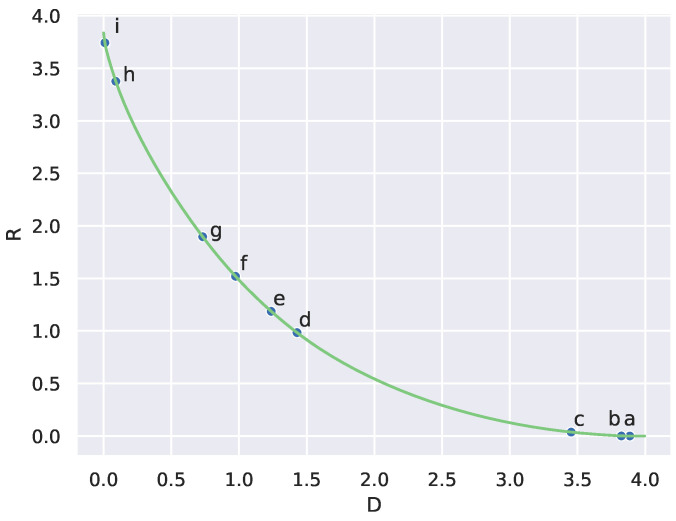
RD curve (in green) and locations of critical codebooks (circles a–i) for 2×2 ON/OFF natural image patches (compare with [80], Figure 9a).

In the language of RD theory, these stochastic Input/Output systems $p_{\beta }\left(y|x\right)$ are called codebooks and they determine—via a rate parameter β—a minimal communication rate *R* for encoding the state *x* as *y*, given a desired average distortion *D*. More specifically, the control parameter t=β indexes a pair $\left(D_{\beta },R_{\beta }\right)$ on the RD function, which separates possible from impossible encodings by a continuous curve in the positive orthant (see Figures 4 and 5).

For example, any lossy compression of a signal class has a particular rate and reconstruction error, which must necessarily lie on or above this curve. Intriguingly, the RD function contains a discrete set of special points $\left(D_{\beta^{*}},R_{\beta^{*}}\right)$ that correspond to critical control parameters $\beta^{*}$ and codebooks $p_{\beta^{*}}\left(y|x\right)$, where some states *y* disappear or reappear in a coding. Intuitively, these are the behavioral phase transitions that must occur when traversing the RD curve from zero to maximal distortion.

In biology, the system *A* could be an organism that lossily encodes a signal through a channel bottleneck. For example, the retina communicates via the optic nerve to the cortex, which is argued to be a several-fold compression of information capture from raw visual input [31]. We may also zoom out in scale to consider collections of organisms. In this setting, the theory of punctuated equilibrium in evolution [35] proposes that species are stable (in equilibrium) until some outside force requires them to change, upon which, species adapt quickly (punctuation). Here, the control parameter might correspond to some environmental variable in the habitat of the species, and sharp phase transitions [36,37] could emerge from some underlying normative dynamics [38,39,40,41,42].

We first validated our approach on classical RD problems with a number of states *n* small enough for comparison with mathematically exact calculations. We demonstrate that the critical $\beta^{*}$ arising from theory match those that correspond to significant peaks in the divergence rate. We also discovered in a large-scale numerical experiment that there is an explicit relationship between counts of these critical control parameters and the number of Input/Output states. Namely, under mild assumptions, we conjectured that there are n−1 critical $\beta^{*}$, corresponding to n−1 critical codebooks $p_{\beta^{*}}\left(y|x\right)$ and critical pairs $\left(D_{\beta^{*}},R_{\beta^{*}}\right)$, for an RD function on *n* states (Conjecture 1). As another application, we show how experiments with our measure shed light on the weak universality conjecture [43], which has implications for efficient systems in engineering and nature.

We next explored several applications in more practical domains. In image processing, for instance, it is commonly desired to encode a picture with a small number of bits that nonetheless represent it faithfully enough for further computation down a channel. In human retina, this is thought to be accomplished by the firing patterns of ON and OFF ganglion neurons, which represent intensity values above or below a local mean, respectively. Using a standard database of natural images [44], we computed the RD function for ON/OFF encodings of small patches and studied the structure of its critical β. We found that our measure uncovers several significant phase transitions for encoding these natural signals. Interestingly, the number of such critical points on the RD function seems to be significantly smaller than that predicted for a generic RD problem. This finding suggests that natural images define a special class of distributions [45] that might be exploited by visual sensory systems for efficient coding [46].

In machine learning, a common problem is to adapt model parameters to achieve optimal performance on a task. Clustering noisy data is one such challenge, and information theory provides tools for studying its solution [33,47]. We found that critical RD codebooks uncovered by the divergence rate can reveal original cluster centers and their count. Another challenge is to store a large collection of patterns in a denoising autoencoder. We examined a specific example of robustly storing an exponential number of memories [48,49] in a Hopfield network [50], given only a small fraction of patterns as training input. We show that as the number of training samples increases, the divergence rate detects critical changes in dynamics, which allow the network to increase performance until generalizing to the full set of desired memories (graph cliques). Thus, we believe that the method of finding criticality described in this paper can be applied to understanding phase transitions in machine learning algorithms. For example, of particular interest is when large language models begin to be able to give the correct answers to certain types of questions [51]. This represents a type of generalization criticality in the space of model parameters.

As a final application, we studied critical phenomena in writing. It has been pointed out that phase transitions arise in the geometry of language and knowledge [52,53,54,55,56,57]. We studied agriculture during the 1800s in the United States using journal articles and uncovered conceptual phase transitions across certain years. In particular, we found that an important shift in written expression occurred during the year 1840. Upon closer examination of the data, there were indeed significant language changes coinciding with the influences of war, religion, and commerce that were occurring at the time.

The outline of this paper is as follows. We give the requisite background for defining divergence rate in Section 2. Next, we explain in Section 3 the findings from applying our measure to various domains such as RD theory, sensory coding, machine learning, and language. We close with a discussion in Section 5 and a conclusion in Section 6.

## 2. Background

We first define a measure of conditional distribution change over an independent control variable, which we call a divergence rate. As the control parameter varies, peaks in this divergence rate can be used to predict critical phase transitions in a system’s behavior. Our methodology is inspired by the work of [54], who used the same measure to compute surprise in a sequence of successive debates of the French Revolution and, hence, which topics tended to gain traction. We also give a brief background on rate–distortion theory, which provides a powerful class of examples to validate our approach and its utility as a tool for discovering new results in the field (e.g., Conjecture 1).

### 2.1. Definition of Measure

Let *X* be a set of data points with underlying distribution $q={\left(q_{x}\right)}_{x\in X}$; that is, the probability of x∈X is given by $q_{x}>0$. Also, suppose for real parameters *t* and each x∈X that there are conditional distributions $p_{t}\left(y|x\right)$ specifying the probability of an output *y* given input *x*. The variable *t* could be a rate–distortion parameter β in RD theory, the size of a training dataset, the number of epochs for estimating a model, or simply time. Where $M_{t}$ is written as *M*, the independent variable should be inferred as *t*. In this work, we restrict our attention to discrete distributions so that *X* is a finite set.

**Definition 1** (Divergence rate)**.**  *The following non-negative quantity measures how much a system changes behavior from t to t+Δt:*

$$M_{t}=\frac{1}{\Delta t}\sum_{x\in X}q_{x}D_{KL}\left(p_{t}\left(y|x\right)∥p_{t+\Delta t}\left(y|x\right)\right).$$

The quantity $D_{KL}\left(u∥v\right):=\sum_{y}u_{y}\log\left(\frac{u_{y}}{v_{y}}\right)$ is the Kullback–Leibler divergence of two distributions *u* and *v*, although other such functions could be used. The divergence rate is a simple proxy for the rate of change of a system’s behavior. In particular, to detect critical *t*, we find local maxima in $M_{t}$ across a range of values of the parameter *t*.

The following lemma validates the intuition that the divergence rate measures phase transitions.

**Lemma 1.**  *Suppose that $p_{t}\left(y|x\right)\neq 0$ for any x,y and that $p_{t}\left(y|x\right)$ is differentiable at t. Then, the divergence rate $M_{t}$ limits to zero as Δt goes to zero.*

**Proof.**  Set $\dot{p_{t}}\left(y|x\right):=\frac{dp_{t}\left(y|x\right)}{dt}$. Consider the quantity $\frac{1}{\Delta t}D_{KL}\left(p_{t}\left(y|x\right)∥p_{t+\Delta t}\left(y|x\right)\right)$ with Δt small for a fixed *x*, which looks like

$$\approx -\frac{1}{\Delta t}\sum_{y}p_{t}\left(y|x\right)\log\left(1+\frac{\dot{p_{t}}\left(y|x\right)}{p_{t}\left(y|x\right)}\Delta t\right)\approx -\sum_{y}\frac{p_{t}\left(y|x\right)}{\Delta t}\frac{\dot{p_{t}}\left(y|x\right)}{p_{t}\left(y|x\right)}\Delta t=-\sum_{y}\dot{p_{t}}\left(y|x\right).$$

Since $\sum_{y}p_{t}\left(y|x\right)=1$, we have $\sum_{y}\dot{p_{t}}\left(y|x\right)=0$, so that the limit of $M_{t}$ as Δt goes to zero is indeed zero.  □

On the other hand, it is easy to verify by its definition that the divergence rate is infinite for any t,Δt such $p_{t}\left(y|x\right)\neq 0$ for all x,y, but for which there is some state *y* with $p_{t+\Delta t}\left(y|x\right)=0$.

### 2.2. Determining Critical Control Parameters

To locate the critical control parameters of an evolving system $p_{t}\left(y|x\right)$, we find all significant local maxima produced by the measure. In practice, numerical imprecision or the presence of noise results in a divergence rate that has several local maxima that likely do not represent critical changes in system behavior. To filter out such false positives, we first normalize the set of divergence rates over samples by subtracting the mean value and then dividing it by the standard deviation. This allows us to filter out peaks that are not significant (for example, peaks ≤α for some α>0). From here onwards, divergence rate will refer to this normalized divergence rate.

In practice, the divergence rate $M_{t}$ is potentially non-concave and since *t* is sampled, we are left with finding peaks in a piecewise linear function. To this end, we utilize a discrete version of the Nesterov momentum algorithm [58] to find significant peaks. We find that the approach is fairly insensitive to hyperparameters when implemented. We provide explicit details of this method and its pseudocode in Section 4.

### 2.3. Rate–Distortion Theory

Rate–distortion (RD) theory ([59], Chapter 10) has frequently been used to analyze and develop lossy compression methods [60] for images [61], videos [62], and even memory devices [63]. It also offers some perspective on how biological organisms organize themselves based on their perceptions of the world around them [64]. For example, rate–distortion theory has been used to understand fidelity–efficiency tradeoffs in sensory decision-making [42] and has also been useful in understanding relationships between perception and memory [65]. At a molecular level, it has been used to study the communication channels and coding properties of proteins [39,66].

Suppose that we have a system with *n* states X={1,…,n} and an input probability distribution $q=(q_{1},\ldots ,q_{n})$ defined on *X*. We seek a deterministic coding of *x* to *y*, with the overall error arising from this coding determined by a distortion function d(x,y)≥0, which expresses the cost in setting *x* to *y* (typically, we assume d(x,x)=0 for all *x*).

Remarkably, provided only with *q* and *d*, the Blahut–Arimoto algorithm [67,68] produces the convex curve of minimal rate R(D) over all deterministic codings with a fixed level of average distortion *D*. For example, when D=0, so that error is not allowed in a coding, the optimal rate is the Shannon entropy H(q) of the input distribution *q*. In general, when D>0, the minimal rate achievable for a coding with average distortion *D* is less than H(q) and is determined by a unique point on the RD curve. This is intuitive as a sacrifice of some error in coding should yield a dividend in a better possible rate.

Although the RD curve determines a theoretical boundary between possible and impossible deterministic coding schemes, the curve itself is determined via an optimization over stochastic codings, which do not directly lend themselves to practical deterministic implementations. Nonetheless, computing the RD curve is still useful for understanding the complexity of a given problem and benchmarking. See Figures 4 and 5 for examples of RD curves in various settings.

Mathematically, the RD curve is parameterized by a real number β that indexes an optimal stochastic coding of *x* to *y* as a codebook or matrix of conditionals $p_{\beta }\left(y|x\right)\geq 0$ (so that $\sum_{y}p_{\beta }\left(y|x\right)=1$). The average distortion is given by $\sum_{x,y}q_{x}p_{\beta }\left(y|x\right)d\left(x,y\right)$. We also set $p_{\beta }\left(y\right)=\sum_{x}q_{x}p_{\beta }\left(y|x\right)$ to be the corresponding distribution for a given output *y*.

It is classical, using Lagrange multipliers, that points on the RD curve arise from solutions to a set of equations in the codebooks and output distributions. For expositional simplicity in the following, we drop β in subscripts and set $w=e^{-\beta }$. We also identify p(y) and p(y|x) with $p_{i}$ and $p_{ij}$, respectively. The governing equations for the RD curve are then given by the following.

**Definition 2.**  *The Blahut–Arimoto (BA) equations for the RD curve are*

(1) $$p_{i}=\sum_{j=1}^{n}q_{j}p_{ij},\;Z_{j}=\sum_{i=1}^{n}p_{i}w^{d_{ij}},\;p_{ij}=p_{i}w^{d_{ij}}/Z_{j}.$$

When the numbers $d_{ij}$ are non-negative integers, solutions to the BA equations form a real algebraic variety, which allows them to be computed symbolically using methods of computational algebra [69].

**Example 1**  (two states). *When n=2, the three equations in (1) translate to these for $p_{1}$ and $p_{2}$:*

(2) $$\begin{array}{cc} p_{1} & =p_{1}\left(\frac{q_{1}w^{d_{11}}}{p_{1}w^{d_{11}}+p_{2}w^{d_{21}}}+\frac{q_{2}w^{d_{12}}}{p_{1}w^{d_{12}}+p_{2}w^{d_{22}}}\right),\\ p_{2} & =p_{2}\left(\frac{q_{1}w^{d_{21}}}{p_{1}w^{d_{11}}+p_{2}w^{d_{21}}}+\frac{q_{2}w^{d_{22}}}{p_{1}w^{d_{12}}+p_{2}w^{d_{22}}}\right). \end{array}$$

More generally, set *W* to be the n×n matrix defined by $W_{ij}=w^{d_{ij}}$. (Note that *W* is invertible near w=0). Then, the BA Equation (1) are easily seen to combine into a single compact equation for any *n* as follows, where $p=(p_{1},\ldots ,p_{n})$:

(3) $$p=p⊙\left[W\left(\frac{q}{W^{⊤}p}\right)\right].$$

Here, ⊙ is the point-wise (Hadamard) product of two vectors, $\left(\frac{u}{v}\right):=\left(u_{1}v_{1}^{-1},\ldots ,u_{n}v_{n}^{-1}\right)$ is the element-wise ratio of the vectors *u* and *v*, and $W^{⊤}$ is the transpose of *W*. Note that for any *p* and *q* in the simplex, the right-hand side of (3) is also in the simplex.

Interestingly, when the BA equations are written in this form, we can identify the RD curve as a fixed-point using Browder’s fixed point theorem [70,71]. We summarize this finding with the following useful characterization.

**Proposition 1.**  *The RD curve is given by the following equivalent objects.*

*1.*  *The minimization of the RD objective function.*
*2.*  *Browder’s fixed-point for the function on the right-hand side of (3).*
*3.*  *Solutions to the BA equations.*

**Proof.**  The equivalence of the first and third statement is classical theory. For the equivalence of the second and the third, consider the map from the product of an interval and the simplex to the simplex given by $f\left(w,p\right)=p⊙\left[W\left(\frac{q}{W^{⊤}p}\right)\right]$. By Browder’s fixed-point theorem, this has a fixed-point given by a curve $p_{w}$, satisfying $f\left(w,p_{w}\right)=p_{w}$, which is Equation (3).  □

When some of the indeterminates $p_{i}$ become zero at some β, then the BA equations still make sense, we simply end up with fewer variables. Such equations involving inverses of linear forms also appear in work on the entropic discriminant [72] in algebraic geometry and maximum entropy graph distributions [73].

When β is large (*w* near 0), optimal codebooks are close to the identity, corresponding to average distortion near zero. In this case, it can be shown directly using Equations (3) that there is an explicit solution [30]. Set 1=(1,…,1) to be the all-ones vector.

**Lemma 2.**  *The exact solution for small w (large β) of the RD function is given by*

(4) $$p=W^{-⊤}\left(\frac{q}{W^{-1}1}\right).$$

**Proof.**  When β is very large, all $p_{i}$ are non-zero. In this case, we may cancel *p* on both sides of Equation (3) to give

$$1=W\left(\frac{q}{W^{⊤}p}\right)\;⟹\;W^{-1}1=\left(\frac{q}{W^{⊤}p}\right)\;⟹\;\left(\frac{q}{W^{-1}1}\right)=W^{⊤}p.$$

The lemma now follows by multiplying both sides of this last equation by $W^{-⊤}$.  □

It turns out that the rate–distortion curve has critical points on it where dramatic shifts in codebooks occur, an observation that was one of our inspirations. For the purposes of this work, these critical $\beta^{*}$ are defined when some $p_{i}$ goes from positive to zero (or visa-versa) as β varies (we remark that in the literature, another equivalent definition is usually used, which is when the derivative of the RD curve has a discontinuity). Note that the third equation in (1) implies that this is when $p_{ij}$ also has such a change. Given this definition, Lemma 1 shows that the divergence rate characterizes these critical points.

The first critical $\beta^{*}$ can be determined using Lemma 2 as follows.

**Corollary 1.**  The first critical $\beta^{*}$ on the RD curve going from β=∞ to $\beta =\beta^{*}$ is the first value of β that makes some entry $p_{i}$ of the vector in Equation (4) equal to zero.

**Proof.**  Equation (4) describes the values of $p_{i}$ along the RD function as long as we are allowed to cancel *p* from both sides of Equation (3). For continuity, the first time a $p_{i}$ becomes zero is governed by when (4) first has this happen.  □

In the next section, we shall continue with our two-state Example 1 above and compare this theoretical calculation from Corollary 1 to the first critical β found by our criticality measure (see Figure 1).

## 3. Applications

We present applications of finding peaks (Section 2.2) in the divergence rate (Definition 1) to the discovery of phase transitions in various domains such as rate–distortion theory, natural signal modeling, machine learning, and language.

### 3.1. Rate–Distortion Theory

Recall that given a distribution *q* and a distortion function *d*, the rate–distortion curve characterizes the minimal rate of a coding given a fixed average distortion. A point on the curve $\left(D_{\beta },R_{\beta }\right)$ is specified by a parameter β and is determined by an underlying codebook $p_{\beta }\left(y|x\right)$. In particular, the setup for RD theory can be seen as a direct application of our general framework with the control parameter t=β.

In the following subsections, we shall validate our approach to finding critical phase transitions by comparing our peak finder estimates of critical β with those afforded by RD theory. In cases where the number of states is small, such as n=2 and n=3, we can compute explicit solutions to the RD Equation (1) and compare them with those estimated by our divergence rate approach (see Figure 1 and Figure 2a,b).

After demonstrating that peaks in the divergence rate agree with theory in these cases, we next turn our attention to exploring what our criticality tool can uncover theory-wise for the discipline. In particular, our experiments suggest new results for RD theory, such as that for a random RD problem, the number of critical $\beta^{*}$ on *n* states goes as n−1 (Conjecture 1). We also use our framework to help shed light on a universality conjecture [43] inspired by tradeoffs in sensory coding for biology (Conjecture 2).

#### 3.1.1. Exact RD on Two States

The case of n=2 states with varying distortion is already interesting. Consider source distribution q=(1/2,1/2) and distortion matrix $d={\left[d_{ij}\right]}_{i,j=1}^{2}$ for fixed a≠0 given by

$$d=\left[\begin{array}{cc} 0 & 1\\ a & 0 \end{array}\right].$$

In this case, using Lemma 2, one can explicitly solve the equations for the output distributions $p_{1},p_{2}$ before the first critical point in terms of β (recall, $w=e^{-\beta }$):

$$p_{1}=\frac{1-2w^{a}+w^{a+1}}{2\left(1-w\right)\left(1-w^{a}\right)},\;p_{2}=\frac{1-2w+w^{a+1}}{2\left(1-w\right)\left(1-w^{a}\right)}.$$

From Corollary 1, the first critical $\beta^{*}$ is determined when one of these expressions becomes zero. One can check that for a>1, $p_{2}$ first has a zero value for some critical β. At this value, $p_{1}$ is automatically determined to be $p_{1}=1$. For a<1, on the other hand, it is $p_{1}$ that becomes zero.

We consider the case a>1, as the other is similar. Our main tool is the generalized version of Descartes’ Rule of Signs [74,75]. This rule says that an expression in a positive indeterminate *w*, such as $1-2w+w^{a+1}$ from above, has a number of positive real zeroes bounded above by the number *s* of sign changes of its coefficients. Moreover, the true number of positive zeroes can only be *s*, s−2, *…*, or s−2⌊s/2⌋.

In our particular setting, we have s=2 so that there can only be two or no positive zeroes. As w=1 is a zero, it follows that there is some other positive number $w^{*}$ making this expression zero. It follows that our first critical point is $\beta^{*}=-\ln\left(w^{*}\right)$. In particular, when a=2, we have

$$\beta^{*}=-\ln\left(\frac{\sqrt{5}-1}{2}\right).$$

Given these explicit calculations from the theory in hand, we would like to compare them with critical parameters determined from significant peaks in the divergence rate.

We summarize our findings in Figure 1. To produce the “measured” points in this plot, we varied the distortion measure using the parameter *a*, computed codebooks parameterized by β with the BA algorithm, and then found critical values of w=exp(−β) at which the divergence rate peaked. At all values of a≠1, the critical value of *w* found by the noisy peak finder matched the corresponding exact value from the theory (the “algebraic” line). Note that near a=1, the critical $\beta^{*}$ approached zero but was not defined there. Also, from the plot, one can guess that $\beta^{*}$ approaches −ln2 (resp. infinity) as *a* goes to infinity (resp. zero). This can also be verified directly from the theoretical calculations above.

#### 3.1.2. Exact RD on Three States

We now consider a slightly more complicated example from Berger [76] with n=3 states. In this case, we assume a varying source q=((1−u)/2,u,(1−u)/2) for a parameter u∈(0,1), but we fix a distortion matrix:

(5) $$d=\left[\begin{array}{ccc} 0 & 1 & 2\\ 1 & 0 & 1\\ 2 & 1 & 0 \end{array}\right].$$

Again using Lemma 2, we find that for large β, the output distributions on the RD curve are given by

(6) $$p=\frac{1}{2{\left(w-1\right)}^{2}}\left[\begin{array}{c} (u+1)w+u-1\\ w^{2}+\left(u-1\right)w+u\\ (u+1)w+w-1 \end{array}\right].$$

For example, when u=1/13, this becomes

$$p=\frac{1}{13{\left(w-1\right)}^{2}}\left[\begin{array}{c} 6-7w\\ 1-12w+13w^{2}\\ 6-7w \end{array}\right].$$

In this special case, one can check that the first critical $w^{*}$ occurs when $p_{2}$ becomes zero; that is, when

$$w^{*}=\frac{1}{13}\left(6-\sqrt{23}\right),\;\beta^{*}=-\ln\left(w^{*}\right).$$

More generally, one can check that as long as $u\leq 3-2\sqrt{2}$, a critical $w^{*}$ is determined from the formula

(7) $$w^{*}=\frac{1}{2}\left(1-u-\sqrt{1-6u+u^{2}}\right).$$

In Figure 2a, we plotted for three different settings of $u=1/13,3-2\sqrt{2},1/5$ the values of the output distribution $p_{2}$ for the second state as a function of β, computed from the general solution (6). On the same plot, we placed two dots on the *x*-axis where we found β representing peaks in the divergence rate in the case of u=1/13. Notice they are located where the output probability of state two went to zero or emerged from zero.

Figure 2b was obtained by plotting the divergence rate as a heatmap against *u* and β. The peak of the bottom convex shape corresponded to the value of $u=3-2\sqrt{2}$, where a discontinuity appeared in the number of critical β given a fixed *u*. Also shown in Figure 2b is a theoretically determined part of this curve, which matched precisely with the estimated lower-right piece determined using only the divergence rate.

#### 3.1.3. Criticality for Generic RD Theory

With these examples as evidence that the empirical divergence rate is able to detect critical β for RD theory, we explored its potential implications for the general case as we increased the number of states *n*.

We next considered random RD problems on *n* states in which the probabilities for *q* were chosen independently from a uniform distribution in the interval [0,1] (and then normalized to sum to one) and the distortion matrices were chosen to have diagonal zero and other entries $d_{ij}$ drawn independently and uniformly in the interval [0,4]. (Our experiments appeared to be insensitive to the particular underlying distributions used to make *q* and *d*).

As shown in Figure 3, we varied *n* and plotted the number of significant peaks in the divergence rate for these random RD problems, averaged over 10 trials. The divergence rate was imperfect and, thus, there were error bars. The accuracy of the divergence rate depended highly on whether the range of β was adequately densely sampled and whether the range of samples included all critical β. It should be noted that this method of finding critical points was an estimate and, therefore, may have indicated large changes in the codebooks where there was no theoretical critical point.

The numerical results shown in Figure 3 suggest the following new conjecture in the field of RD theory.

**Conjecture 1.**  Generically, there are n−1 critical $\beta^{*}$ along the rate–distortion curve as a function of the number of states n.

Here, the word “generic” is used in the sense that an RD problem on *n* states with data distribution *q* and distortion matrix *d* “chosen at random” will have this number of critical points. More precisely, if $d_{xy}=d\left(x,y\right)$ and $q_{x}$ are non-negative indeterminates with $q_{x}$ on the simplex, then outside of a set of measure zero, the number of critical β on the RD curve determined by *d* and *q* will be exactly n−1.

#### 3.1.4. Weak Universality

Consider the case where all *n* states have the same probability $q_{x}=1/n$ and two distortion matrices $d_{1}$, $d_{2}$ have entries drawn from the same distribution, for example, uniform in [0,1]. Then, a conjecture in RD theory, called weak universality, says that the RD curves for $d_{1}$ and $d_{2}$ lie on top of each other for large *n*.

**Conjecture 2**  (Weak universality [43])**.** *Distortion matrices with entries drawn i.i.d from a fixed distribution ϕ define the same limiting RD curves as the number of states goes to infinity.*

To illustrate weak universality in Figure 4, we took two randomly drawn distortion matrices $d_{1}$ and $d_{2}$ and plotted their RD curves, computed using the BA algorithm. We then plotted the critical points found from determining peaks in the divergence rate on each of the two RD curves. As predicted by Conjecture 2, the RD curves are close. However, notice that the two sets of critical points do not align with each other.

This experiment suggests that we can rule out a line of attack for proving weak universality that attempts to show critical points on the RD curve that also coincide with each other. In particular, this stronger form of the conjecture is likely not true.

### 3.2. Natural Signals

Discrete ON/OFF encodings of image patches have been shown to contain much perceptual information [77], and Hopfield Networks trained to store these binary vectors can be used for the high-quality lossy compression of natural images [78,79].

It was also observed in [80] that these ON/OFF distributions of natural patches exhibited critical $\beta^{*}$ at a few special points and codebooks along the RD curve. Applying our noisy peak finder to the divergence rate on the codebooks at rate parameters β, we were able to discover all the critical codebooks that were previously found by hand, as well as four more that had deviations in microstructure. We remark that it is also possible that some of these extra critical codebooks found may have arisen from imperfections in the tuning of the peak finder.

To obtain Figure 5, two-by-two pixel patches *x* were ternarized by first normalizing the pixel values within each patch to have variance one, and then each normalized value *v* was mapped to a ternary value *b* via the following rule:

$$b=\left\{\begin{array}{cc} (0,1), & \mathrm{if}\;v<-0.5,\\ (0,0), & \mathrm{if}\;-0.5\leq v\leq 0.5,\\ (1,0), & \mathrm{if}\;v>0.5. \end{array}\right.$$

We used as source *q* the probability distribution over this ternary representation of patches, and the Hamming distance between two binary vectors specified the distortion matrix. Codebooks along the RD curve were then used as conditional distributions and peaks in the divergence rate were plotted as the nine blue dots.

**Figure 6:**
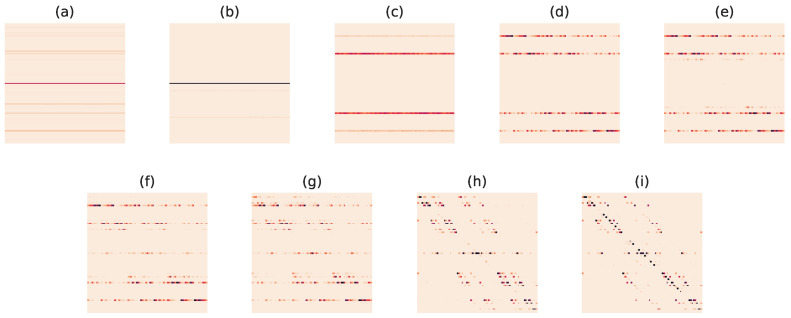
Natural image patch codebooks at critical β from Figure 5 estimated using the divergence rate (compare with [80], Figure 9c). Codebook matrices displayed in (**a**–**i**) correspond to the circles a–i on the RD curve in Figure 5, with darkest intensities indicating the value 1 and lightest, 0.

In Figure 6, we display the codebooks that arose from the critical $\beta^{*}$ found using the divergence rate. These codebooks correspond to the lettered points in Figure 5.

It is interesting to note that there were an order of magnitude fewer critical codebooks estimated than would have been expected by Conjecture 1. This suggests that this set of natural signals does not represent a random or generic RD problem, indicating extra structure in the signal class.

### 3.3. Machine Learning

We considered a warm-up problem on 18 states to test the divergence rate on the problem of clustering. The data contained three disjoint groups, each with six states, that had low intra-cluster distortion but high inter-class distortion.

**Figure 7:**
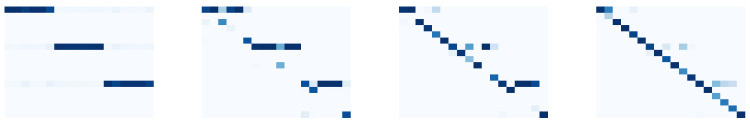
Critical codebooks for clusters corresponding to critical points determined by peaks in the divergence rate. Each column is the conditional distribution given an input (increasing light to dark).

We applied the noisy peak finder to the divergence rate of codebooks parameterizing the RD curve for this setup and plotted a few of the interesting critical codebooks in Figure 7. We noticed that critical codebooks coded for when the cluster centers were found and when the code progressively broke away towards the identity codebook.

**Figure 8:**
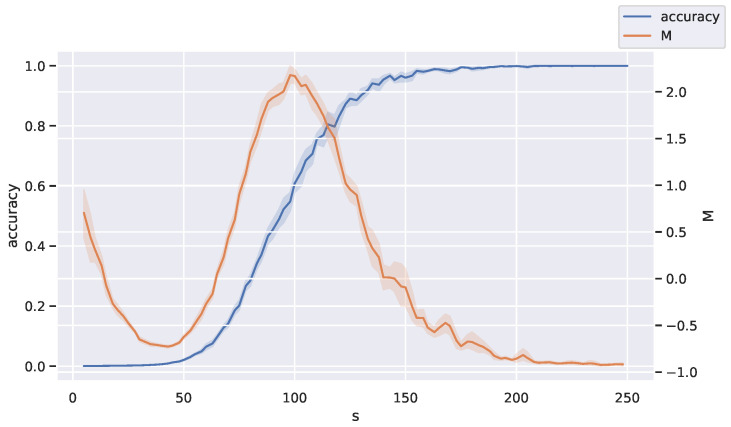
Peaks in the divergence rate *M* are signatures of phase transitions in performance accuracy for Hopfield recurrent neural networks as the number of training samples *s* increases.

Our next machine learning example came from the theory of auto-encoding with Hopfield networks. First, we determined networks to store cliques as fixed-point attractors (memories) using Minimum Energy Flow (MEF) learning, as in [48,49]. Then, we looked for peaks in the divergence rate as networks were trained with different numbers of samples *s*. Our hypothesis was that as *s* was varied, there would be some critical *s* where the nature of the network dynamics changes drastically. For the clique example in Figure 8, the possible data consisted of binary vectors representing the absence or presence of an edge in a graph of size 16, with each sample being a clique of size 8.

By randomly sampling a different set of training data for each trial, we obtained a codebook for each number of training samples as the deterministic map on binary vectors given by the dynamics of the Hopfield network. Specifically, the codebook $p_{s}\left(y|x\right)$ was the 0/1-matrix of the output of the dynamics determined by the association x↦y of the *s*th network; that is, each map formed a conditional matrix entry $f_{s}\left(y|x\right)$ that was $\frac{1}{|X|+1}$ if the network dynamics took state *x* to *y* and zero otherwise, where |X| is the total number of cliques. We used one output state to code for all non-cliques, leading to |X|+1 in the denominator.

We then plotted the accuracy (proportion of cliques correctly stored by the network) in blue and the normalized divergence rate in orange against *s*. We averaged this over 10 trials in the experiment. A significant peak in the divergence rate as a function of *s* can be observed in Figure 8. This suggests a critical sample count for auto-encoding in the Hopfield networks trained using MEF. Note that this peak appeared to occur when exactly half of the test data were accurately coded.

The fact that signatures of criticality in self-organizing recurrent neural networks arise during training is also corroborated by [81]. It would be interesting to further explore the changes that happen near the critical sample count shown in Figure 8.

### 3.4. Language

**Figure 9:**
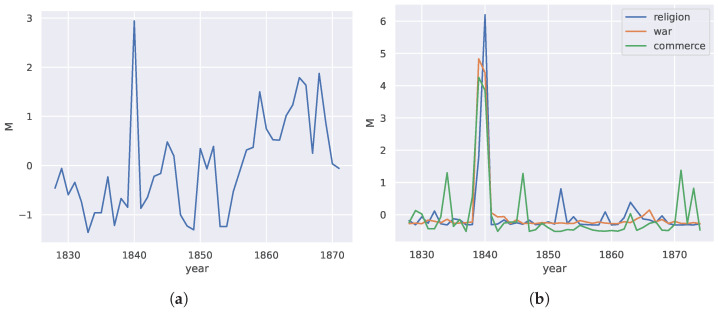
(**a**) Normalized plot of divergence rate for a horticultural journal corpus against time. (**b**) Peaks along time in the corpus associated with the indicated words.

In [55], the possibility of punctuated equilibrium and criticality in language evolution was studied using a similar approach based on the divergence rate. Naively using the divergence rate on raw word frequency distributions over time, we can also obtain a measure of language change over time. In this case, the divergence rate is over a single condition (fixed topic). We show that peaks are also observed in this measure of a language dataset. Figure 9a indicates that critical changes are not only common but a natural part of the way humans solve problems in communication.

For the language example, the codebook was conditioned on a single state, which was the topic of the dataset, namely, agriculture in America. This meant that we only considered the distribution of words of one topic over time as the independent variable *t*. The divergence rate was thus applied over a single state in the language concept space.

To discover what the underlying cause of this large change could be, we clustered the words in the corpus using Word2Vec [82] and found several interesting clusters. We then chose semantically similar words from those clusters, which represented concepts in war, religion, and commerce. Again, by conditioning on words from these subtopics, we used the divergence rate to obtain the changes in the probability distributions of these clusters individually in Figure 9b. We noticed that peaks occurred exactly in the year of the most significant peak in Figure 9a.

Investigating possible reasons why this might be the case, we looked into the history of agriculture in the United States of America. In [83], Frolik mentioned that many innovations and technologies were brought to bear on the American agriculture industry (such as the cast iron plow, manufacturing of drills for sowing seeds, and horse-powered machines for harvesting grain) around the years leading up to and including the 1840s, which led to a booming agricultural industry. This possibly accounts for the peak in the usage of words related to commerce.

## 4. Methods

The measure $M(f_{t})$ is the sum of the distance over x∈X between probability distributions *f* conditioned on *x* at different points *t* and t+Δt. It tracks the change in *f* over *t*. In our experiments, we used the Kullback–Leibler (KL) divergence for *D*.

$$M\left(f_{t}\right)=\frac{1}{\Delta t}\sum_{x\in X}D\left(f_{t}\left(y|x\right),f_{t+\Delta t}\left(y|x\right)\right)$$

The value of *t* here could be replaced by some independent variable, such as β (in the case of the RD curve), the size of training dataset, the number of epochs (for training a model), or time. The codebook of a model *f* (for a discrete state space) is a distribution conditioned on each input state. For a set of models with random initializations, we normalized the distribution of the maps with respect to the inputs over all the maps. The measure over *t* was then obtained by taking the distance between pairs of consecutive codebooks. Critical phase transitions were then where peaks occurred along this 1-dimensional signal.

The measure $M(f_{t})$ is potentially non-concave and the variable *t* is usually sampled at discrete intervals, thus producing a piecewise linear function. Thus, to detect local maxima in the measure that represent points in *t* where the measure changes significantly (a critical point in *t*), we needed a noisy peak finder, as there may have been spurious local maxima that did not correspond to any critical change in behavior. To this end, we developed a discrete version of the Nesterov momentum algorithm [58] that we encapsulate in Algorithms 1–4.

Given a distortion measure (non-negative matrix describing costs of encoding an input state with an output state), the rate–distortion curve describes the minimum amount of information required to encode a given distribution of symbols at a level of distortion specified by the gain parameter β. There is a corresponding codebook at any β that describes a distribution over all symbols *X* for each symbol *x* with which to optimally code for *x*. This allowed us to directly use the measure, using β as the independent variable *t*.
**Algorithm 1** Noisy local maxima finder**Require:** List of 2D points *p*    Step size η    Momentum μ    Convergence threshold ϵ**Ensure:** *p* is sorted by increasing x values    ϵ>0    q← Compute potential local maxima with **Find All Local Maxima** with input *p*    u← points in *p* with $p_{i}.y\leftarrow -p_{i}.y$    r← Compute local minima with **Find All Local Maxima** with input *u*    i←1    $\theta \leftarrow r_{i}.x$    o← new empty set of 2D points    **while** $\theta \neq r_{n}.x$ **do**         θ← compute next local maxima with **Find Next Local Maxima** with inputs *p*, *q*, θ, η, μ and ϵ         Add θ to *o*         **for** j=i+1,i+2,…,r.length **do**               **if** $r_{j}.x-\theta >0$ **then**                    i←j                    $\theta \leftarrow r_{i}.x$                    break               **end if**         **end for**    **end while**

**Algorithm 2** Find All Local Maxima

**Require:**

    List of 2D points *p*
    o← new empty list of 2D points
    **for** i=2,3,…,p.length−1 **do**
           **if** $p_{i-1}.y<p_{i}.y>p_{i+1}.y$ **then**
                Add $p_{i}$ to *o*
           **end if**
    **end for**
    return *o*


**Algorithm 3** Find Next Local Maxima

**Require:**

    List of 2D points *p*
    List of 2D local maxima points *q*
    Current x-value θ
    Step size η
    Momentum μ
    Convergence threshold ϵ
    $\theta_{prev}\leftarrow \infty$
    $\phi_{prev}\leftarrow 0$
    ϕ←0
    **while** $abs(\theta -\theta_{prev})<\epsilon$ **do**
         $\theta_{prev}\leftarrow \theta$
         $\Delta_{\theta }\leftarrow$ compute gradient with **Piecewise gradient** with input *p* and θ
         $\phi_{prev}\leftarrow \phi$
         $\phi \leftarrow \theta +\eta \Delta_{\theta }$
         $\theta \leftarrow \phi +\mu (\phi -\phi_{prev})$
    **end while**
    o←1
    **for** i=1,2,…,q.length **do**
         **if** $abs\left(\theta -q_{i}.x\right)<abs\left(\theta -q_{o}.x\right)$ **then**
              o←i
         **end if**
    **end for**
    return $q_{o}.x$


**Algorithm 4** Piecewise gradient

**Require:**

    List of 2D points *p*
    Point to compute gradient at *t*
    o←1
    **for** i=2,3,…,p.length−1 **do**
           **if** $abs\left(t.x-p_{i}.x\right)<abs\left(t.x-p_{o}.x\right)$ **then**
                o←i
           **end if**
    **end for**
    $g\leftarrow \frac{p_{o+1}.y-p_{o}.y}{p_{o+1}.x-p_{o}.x}$
    return *g*


For the simple two-state rate–distortion example in Figure 1, we varied the distortion measure using a parameter *a* and found the critical value of y=exp(−β) at which the measure peaked. At all values of *a*, the critical value of β found by the noisy peak finder matched the analytic critical value of β. Note that there was no analytic critical value of β at a=1 and, thus, there was a gap in the plot.

For the three-state case proposed by Berger in Figure 2a,b, the distortion matrix was held constant and we varied the probability distribution *q*. As an example, Figure 2a shows the marginal probability of state 2 at $u=\frac{1}{13}$, and the points show where the peak finder detected a critical change, which were visually exactly where the marginal probability went to or emerged from 0. Figure 2b is a plot of the measure as a heatmap against *u*, β.

To arrive at Figure 3, we generated a random distortion matrix for a uniform distribution on *k* states for each trial. We varied *k* and plotted the number of critical β against *k* for the RD curve on this setup. We performed 10 trials to obtain this plot.

To illustrate weak universality in Figure 4, we took two distortion matrices D1 and D2 drawn from the same distribution (uniform in [0,1]). We then plotted the critical points found by the measure on the RD curve.

To test this measure on the coding of clusters, we consider an 18-state distribution, with disjoint groups of six states that have low intra-cluster distortion, but high inter-class distortion. We then apply the noisy peak finder to the measure on the RD curve for this setup and plot some of the interesting critical codebooks in Figure 7.

For the clique example in Figure 8, the data is binary vectors representing absence or presence of an edge in a graph, with each sample being a clique of size $\frac{v}{2}$, where *v* is the number of nodes in the graph, taken to be 16 in our experiment. The distortion matrix is computed using Hamming distance. By randomly sampling a different set of training data for each trial, we obtain a codebook for each number of training samples. We then plot the accuracy (proportion of cliques correctly stored exactly by the network) in green and the normalized measure in purple against the number of training samples.

For the natural image patch example in Figure 5, the data were a ternarized form of two by two pixel patches. Each pixel was normalized and then represented by two bits. If the normalized value of the pixel was greater than a threshold value α=0.5, it was represented as (0,1); if it was less than −α=−0.5, it was represented by (1,0); otherwise, it was represented by (0,0). Thus, in a two-by-two patch, there were $3^{4}=81$ states. Again, the distortion matrix was the Hamming distance between two bit vectors. We obtained codebooks along the RD curve and plotted points where critical transitions were found.

For the language example, the codebook was conditioned on a single variable, which was the topic of the dataset: agriculture in America. This meant that we only considered the distribution of words of one topic over time as the independent variable *t*. The measure was thus over a single state in the language concept space.

## 5. Discussion

In this section, we discuss implications of our criticality measure. Our experiments show that criticality in system behavior appears to be relatively common, as predicted by RD theory. In this paper, we have shown that optimization procedures can also lead to critical transitions in function behavior and that in natural processes such as language evolution, such critical changes can also occur. It could be that these processes find encodings that are close to rate-optimal, as there may be a significant discontinuity in the rate of change $\frac{dR}{dD}$ of encoding cost with respect to encoding error at these points.

This analysis, however, only accounts for the critical points on the RD curve. The critical points of a process would depend on the details of the process itself, and we can only expect the critical points to line up with the ones on the RD curve if the process is efficient enough to follow the RD curve near its critical points. It is possible to detect these discontinuities in $\frac{dR}{dD}$, though measuring it would require far more samples.

Another point that can be made is that criticality does not necessarily manifest as points but as manifolds. In Figure 2b, a 1-dimensional critical manifold is observed in *u*. This is akin to how the boiling point of a substance is a function of pressure and temperature. Further work could involve studying the behavior of these manifolds.

Observing the codebooks at the critical points in order of increasing distortion, we found that the codebook broke away further from encoding the distribution as the identity function. Further work could be to observe the microstructure of codebooks at the critical points to try to understand which states are chosen to reduce the cost of coding perfectly at each critical point.

There are several direct implications for critical behavior, some of which were outlined by Sims in [84]. One open question is how to apply these ideas to deep learning [85] to minimize a typically squared error loss. One insight is to choose models and normative principles that have critical signatures [65] (working memory). Given that optimal continuous codings are discrete [80,86,87,88], it is not that surprising for criticality to arise from near-optimal solutions to normative principles applied to information processing. Furthermore, it has been observed that generalization beyond overfitting a training dataset, such as that seen in grokking by large language models, is related to the double descent phenomena [28], which has been shown to be dependent on multiple factors [89]. These relationships could be further explored using the method described in this paper by, for example, estimating the $D_{KL}$ of more complex distributions using [90].

Further work would involve continuous distributions, which require different treatment to obtain the RD curves [80], and the use of different probability distribution distance metrics. Other algorithms for mapping discrete spaces could also be analyzed with the techniques developed in this work.

Finally, it would be interesting to investigate how other criticality measures relate to the information theory-inspired divergence rate that is presented here. For instance, in the field of reinforcement learning, it can be useful to identify critical choices of an agent’s actions [91] when performing a task. As another example, in graph analyses, it is often crucial to identify nodes that are critical for optimal flows on the graph. In this case, a maximum entropy approach using path trajectories can be found in [92]. Adapting these ideas to our setting will be explored in future work and is beyond the scope of this paper.

## 6. Conclusions

Our initial hypothesis was that an information processing system that compresses and reinterprets information into a useful form usually has critical transitions in the way the information is transformed as some control parameter of the system is tweaked. This was confirmed with the use of the divergence rate—the measure we developed to track the change in the encodings along the control parameter—and a noisy peak finder, which helped to identify critical points. We believe that this may provide fertile ground for further research into critical phenomena in other areas such as the behavior of learning algorithms.

## Data Availability

No primary research software or code have been included. This study was carried out using publicly available data from “Van Hateren’s Natural Image Database” at https://github.com/hunse/vanhateren (accessed on 7 November 2024), and from “American Farmer; Devoted to Agriculture, Horticulture and Rural Life 1819–1897” at https://archive.org/details/pub_american-farmer-devoted-to-agriculture-horticulture (accessed on 7 November 2024).

## Abbreviations

The following abbreviations are used in this manuscript:

RD	rate–distortion
$D_{KL}$	Kullback–Leibler divergence
MEF	minimum energy flow
